# 

**Published:** 1891-10-31

**Authors:** 


PRICE ONE PENNY.

No. 266, Vol. XI.—October 31st, 1891.

[Registered at the General Post Office as a Newspaper for transmission in the United Kingdom and Abroad.]

WITH EXTRA NURSING SUPPLEMENT.

Per Annum, 6s. 6d.; post free

Monthly Parts, 6d.; post free 8d.

Ditto per Annum, 8s., post free.

### THE HOSPITAL

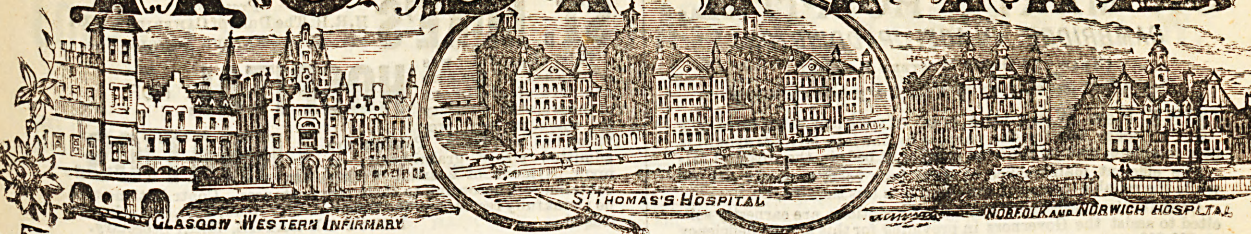

A WEEKLY JOURNAL OF

### Science, Medicine, Nursing and Philanthropy.

SATURDAY, OCTOBER 31st, 1891.

Nurses' Grievances at Glasgow . . . . . . . 49

Passing Topics.—Harvest Festivals . . . . . . 50

The Doctor's Armchair.—The Queen and the Cold Weather 51

Some Scientific Aspects of Insanity. II.—Brain and Spinal Cord . . . . . . . . . . 52

The Hospitals Association . . . . . . . . 53

Everybody's Page . . . . . . . . . 53

Notes and News . . . . . . . . . 54

General Charities . . . . . . . . . 55

Scraps and Gleanings . . . . . . . . 55

Annotations.—Brain Rust—Sleeplessness and Suicide—Dangerous Practitioners . . . . . . . . 56

The Practitioner's Mirror and Retrospect—

Medicine.—V.—New Methods of Treatment in Phthisis, 57; Obesity: Its Causes and Treatment. II.—Treatment . 58

Ophthalmology.—Amblyopia from Errors of Refraction . 53

The Practitioner's Bookshelf.—Medico-Legal Experience in Calcutta . . . . . . . . . 59

New Drugs, Appliances, and Things Medical.—Frame Food . . . . . . . . . . . 59

Editor's Letter Box.—The Hospitals Association . . . 59

The Student of Medicine.—Appointments—Vacancies—Notes on Instruments—Students' Medical Societies—Scholarships and Prizes—Pass Lists—Events of the Month of October

EXTRA SUPPLEMENT—THE NURSING MIRROR.

En Passant.—Saint Helens—Short Items—Govan Goes Forward — A Bournemouth Bazaar — Entertainments for Nurses—The Name of "Nurse"—Blackburn Infirmary . . . . . . . . . xxv

Glasgow Infirmary Nurses . . . . . . . xxvi

Blackburn Nurses' Home . . . . . . . xxvi

Appointment . . . . . . . . . xxvi

A Nurse in Natal . . . . . . . . . xxvi i

Presentations . . . . . . . . . xxviii

Everybody's Opinion.—Nurses' Food—Nurses as Patients . . . . . . . . . . xxix

Notes and Queries . . . . . . . . xxix

A Heroine Discovered . . . . . . . . xxix

A Bed for a Sick Nurse . . . . . . . xxix

Amusements and Relaxation . . . . . . xx x

Off Duty.—From Pharmacy to Farming—(*concluded*) . . xxx

